# IU1 suppresses proliferation of cervical cancer cells through MDM2 degradation

**DOI:** 10.7150/ijbs.47999

**Published:** 2020-09-16

**Authors:** Liu Xu, Jing Wang, Xiaoning Yuan, Shuhua Yang, Xiaolong Xu, Kai Li, Yanqi He, Lei Wei, Jingwei Zhang, Yihao Tian

**Affiliations:** 1 Department of Pathology and Pathophysiology, Hubei Provincial Key Laboratory of Developmentally Originated Disease, School of Basic Medical Sciences, Wuhan University, Wuhan, Hubei 430071, P.R. China.; 2 Department of Pathology, Wuhan No. 1 Hospital, Tongji Medical College, Huazhong University of Science and Technology, Wuhan, Hubei 430022, P.R. China.; 3 Department of Breast and Thyroid Surgery, Zhongnan Hospital of Wuhan University, Hubei Key Laboratory of Tumor Biological Behaviors, Hubei Cancer Clinical Study Center, Wuhan, Hubei 430071, P.R. China.; 4 Department of Human Anatomy, School of Basic Medical Sciences, Wuhan University, Wuhan, Hubei 430071, P.R. China.

**Keywords:** cervical cancer, USP14, MDM2, IU1, UPS

## Abstract

Previous studies have demonstrated that the antitumor potential of IU1 (a pharmacological compound), which was mediated by selective inhibition of proteasome-associated deubiquitinase ubiquitin-specific protease 14 (USP14). However, the underlying molecular mechanisms remain elusive. It has been well established that *mdm2* (Murine double minute 2) gene was amplified and/or overexpressed in a variety of human neoplasms, including cervical cancer. Furthermore, MDM2 is critical to cervical cancer development and progression. Relatively studies have reported that USP15 and USP7 stabilized MDM2 protein levels by removing its ubiquitin chain. In the current study, we studied the cell proliferation status after IU1 treatment and the USP14-MDM2 protein interaction in cervical cancer cells. This study experimentally revealed that IU1 treatment reduced MDM2 protein expression in HeLa cervical cancer cells, along with the activation of autophagy-lysosomal protein degradation and promotion of ubiquitin-proteasome system (UPS) function, thereby blocked G0/G1 to S phase transition, decreased cell growth and triggered cell apoptosis. Thus, these results indicate that IU1 treatment simultaneously targets two major intracellular protein degradation systems, ubiquitin-proteasome and autophagy-lysosome systems, which leads to MDM2 degradation and contributes to the antitumor effect of IU1.

## Introduction

Cervical (uterine cervix) cancer, an increasing threat to women worldwide, is considered as a heterogeneous group of neoplasms originating from cells in the cervix uteri. The clinical effects of cancer treatments such as chemotherapy and radiation therapy vary widely from one patient to the next 1. Thus, it is urgent to find new targets and drugs for the treatment option for cervical cancer.

Recent studies have identified E3 ubiquitin ligase MDM2 (Murine double minute 2) as a novel therapeutic target in cervical cancer 2. Aberrant MDM2 protein expression is documented in a wide variety of human tumors and is thought to be due to gene amplification, transcriptional as well as post-translational regulation 3. Evidences have shown the involvement of proteasome system (Ubiquitin-proteasome system, UPS) in MDM2 degradation 3-5. Indeed, several Deubiquitinating Enzymes (DUBs), such as USP15 and USP7, have been reported to regulate MDM2 expression or transcriptional activity 6-10.

IU1 is a selective pharmacological inhibitor of deubiquitinating enzyme USP14, which inhibits chain trimming and degradation of ubiquitinated proteins 11. Several other proteins implicated in proteotoxic mechanisms-Tau, TDP-43, ATXN3, and glial fibrillary acidic protein (GFAP)-were similarly depleted from mouse embryonic fibroblasts (MEFs) by IU1 11. Due to its anti-proteotoxic properties, IU1 has been proposed as a candidate treatment of neurodegenerative disorders 12-14. Besides, IU1 also presents antitumor properties 15. However, the antitumor function of IU1 and the underlying molecular mechanism in cervical cancer remains unestablished.

Here we demonstrated that IU1, as a pharmacological deubiquitinating enzyme USP14 selective inhibitor, dramatically decreased MDM2 expression, accompanied by blocking G0/G1 to S phase transition, reducing cell growth and triggering cell apoptosis in cervical cancer cells. We propose that IU1 regulates MDM2 protein levels by a post-translational mechanism. The proteasome system UPS and the autophagy system, are the two primary pathways in intracellular protein degradation 16-18. Here, we showed that IU1 treatment promoted UPS function and activated autophagy. Thus, the present studies support the notion that USP14/MDM2-mediated activation of the UPS and autophagy contributes to the antitumor effects of IU1 in cervical cancer cells through MDM2 degradation.

## Materials and methods

### Plasmid constructs

The cherry-MDM2 and pFlag-USP14 vectors were constructed in this experiment. USP14 and MDM2 primers were designed by Primer Premier 5, and the primers sequence were: USP14-F: 5'-CCCGGATCCCCGCTCTACTCCGTTACTG-3' and USP14-R: 5'-AGAGAATTCCTGTTCTTTTTCTCTTCC-3'; MDM2-F: 5'-AAAGAATTCATGGTGAGGAGCAGGCAAAT-3' and MDM2-R: 5'-GGTGGATCCCCGGGGAAATAAGTTAGCACAAT-3'. The sequences were inserted into the cherry and pCMV-Flag vectors, respectively. To meet the experimental needs, the overexpression vector and shRNA knockdown vector of USP14 were constructed. Full length coding sequence of USP14 was amplified by PCR and cloned into pLVX-puro vector with the restriction sites EcoR1 and BamH1. The sequences were as follows: pLVX-USP14-HA-F1: 5'-CGCGAATTCGCCACCATGCCGCTCTACTCCGTTACTG-3'; pLVX-USP14-HA-R1: 5'-CGCGGATCCTTAAGCGTAATCTGGAACATCGTATGGGTACTGTTCACTTTCCTCTTCCAT-3'. The shRNA of USP14 was purchased from the Sigma Company, and the efficiency of knockdown was confirmed. The shRNA was ligated into pLKO.1-EGFP-Puro vector with the restriction sites EcoR1 and Age1. The sequences were as follows: shUSP14-F2: 5'-CCGGCGCAGAGTTGAAATAATGGAACTCGAGTTCCATTATTTCAACTCTGCGTTTTTG-3', shUSP14-R2: 5'-AATTCAAAAACGCAGAGTTGAAATAATGGAACTCGAGTTCCATTATTTCAACTCTGCG-3'. Above-mentioned plasmids were sequenced and confirmed.

### Cell culture and transfection

The human cervical cancer HeLa and SiHa cells were obtained from the Department of Pathophysiology, Basic Medical College of Wuhan University (Wuhan, China). Cells were cultured in DMEM (Hyclone, USA) medium supplemented with 10% FBS (HyClone; GE Healthcare) and antibiotics (1% penicillin G, 1% streptomycin) at 37 °C in a humidified incubator supplemented with 5% CO_2._ Twenty-four hours before transfection, 1.0×10^5^ cells were plated in 2000 μL of growth medium without antibiotics in 6-well plates. The cells were transfected using Lipofectamine 2000 reagent (Thermo Fisher Scientific, USA) according to the manufacturer's protocols.

### Cell proliferation assays

CCK-8 assay (CK04, Dojindo, Japan) was used to detect cell proliferation. The cervical cancer HeLa and SiHa cells were plated in 96-well plates at a concentration of 1×10^4^ per well and incubated for 24 h. Then the cells were treated with a range of concentrations (as indicated in the figure) of IU1 for 24 h. After DMEM (Hyclone, USA) was thoroughly mixed with 1/10 volume of CCK-8, 100 μL mixture was added to each well. Then, the cells were incubated for 2 h at 37 °C. Besides, the inhibition rate of 100 μM IU1 on cell proliferation was examined at 0 h, 12 h, 24 h, and 48 h. The absorbances at 450 nm were measured with an ELISA plate reader (Infinite® 200 PRO, TECAN, Männedorf, CH). The viability of IU1-treated cells was calculated by comparing to vehicle-treated cells, which were arbitrarily assigned 100%. All experiments were repeated three times.

### Clone formation assay

HeLa and SiHa cells treated with different concentrations (as indicated in the figure) of IU1 were plated on a 6-well plate at 1×10^3^ cells per well, and control group was added with an equal volume of DMSO (Amresco, USA). After cultured at 37 °C in a 5% CO_2_ incubator for one week, the clones were stained with crystal violet (0.1%, m/v). Finally, the clones were counted, and the images were captured by the inverted fluorescence microscope (Olympus, Japan). All experiments were repeated three times independently. Clone formation rate = (number of clones versus number of inoculated cells) × 100%.

### Cell migration assay

The two-dimensional migration ability of HeLa and SiHa cells was detected by wound healing assays. HeLa cells were plated (5×10^5^ cells/mL) in a 6-well plate. After the cells were starved for 12 h in low serum, a wound was made by a pipette tip (200 μL). Cells were washed with phosphate buffer saline (PBS), and different concentrations of IU1 (0 μM, 100 μM) with serum-free DMEM was added to each well. Cell wound images were taken at 0 h, 24 h, and 48 h to examine wound healing. The three-dimensional cell migration was detected using Transwell assay. HeLa or SiHa cells were put in the upper transwell chamber. Add 500 μL of complete medium containing 10% serum to the lower chamber. The cells were migrated toward the serum gradient for 24 hours. The migrated cells were fixed by 4% paraformaldehyde and stained with 1% crystal violet. Finally, the migrated cells were observed under a phase-contrast microscope. Five random regions were counted for each experiment.

### Apoptosis assay and cell cycle assay

Apoptosis was detected by flow cytometry using the Annexin V-FITC/PI Apoptosis Detection Kit (Liankebio, Hangzhou, China). The trypsin-digested cells were washed twice with PBS, stained with annexin V-FITC/PI for 15 min, and protected from light at room temperature. Detection was then performed by flow cytometry (FACScan, BD Biosciences).

The cell cycle was detected by flow cytometry using PI staining. The cells were first digested with trypsin and collected, then fixed at 4 °C in 70% ethanol overnight. The cells were suspended in 5 mg/mL propidium iodide and 100 μg/mL RNase A (prepared in PBS) and incubated at 4 °C for 30 minutes at the next day. Finally, the cell cycle was analyzed by flow cytometry.

### Western blot

The protein was extracted with RIPA lysate and quantified with BCA Protein Assay Kit (Beyotime Biotechnology Co, Jiangsu, China). The protein was bound to the PVDF membrane by transmembrane after electrophoretic. After blocking with 2% BSA, the PVDF membrane was incubated overnight at 4 °C with the primary antibodies, including p53 (Abcam, ab26, 1:1000), LC3 (Abcam, ab128025, 1:1000), p62 (Protein Tech, 18420-1-AP, 1:1000), MDM2 (Santa Cruz Biotechnology, sc-13161, 1:1000), ubiquitin (Abcam, ab7780, 1:1000), USP14 (Abcam, ab192618, 1:1000), HA-tag (Proteintech Group, 51064-2-AP, 1:3000), Caspase-3 (Cell Signaling Technology, catalog no. #9664, 1:1000), Caspase-9 (Cell Signaling Technology, catalog no. #9505, 1:1000), Caspase-8 (Cell Signaling Technology, catalog no. #9496, 1:1000), GAPDH (Santa Cruz Biotechnology, sc-32233, 1:5000). The membrane was subsequently incubated with the corresponding secondary antibody (dilution of 1:1000) and finally detected by ECL reagents (Tanon, Shanghai, China) at chemiluminescence imaging system (Tanon, Shanghai, China).

### Immunofluorescence and co-immunoprecipitation

The cells were inoculated into the cell climbing tablets and fixed with 4% paraformaldehyde. Then the cells were sealed with goat serum at room temperature for 30 min. After absorbing the blocking solution, the crawler was incubated with the diluted first antibody overnight at 4 °C. Diluted fluorescent second antibody was added to avoid light for 1 h. Tablets were sealed with anti-fluorescence attenuation tablets and the image were collected under a fluorescence microscope.

For the co-immunoprecipitation assays, appropriate amount of cell lysis buffer was added to the cells. The supernatant was obtained. A small amount of protein solution was taken for Western blot analysis, and the remaining solution was incubated with the corresponding antibody at 4 °C overnight. Then the solution was added with 10 μL pretreated protein A agarose beads, incubated slowly for 4 h at 4 °C. After immunoprecipitation, the supernatant was washed with 1 ml pyrolysis buffer. Finally, the immunocomplexes solution was suspended with SDS buffer and boiled for 5 min. Western blotting analysis was performed as we described above.

### Statistical analysis

Statistical analysis was performed using software Graph Pad Prism 5. Each set of experiments was repeated at least three times, independently. Results are presented as the mean ± standard error of the mean. Statistical significance between groups was tested using *t*-test. *P*<0.05 indicates a statistically significant difference.

## Results

### IU1 treatment negatively regulates the proliferation of HeLa cells

**Figure 1:**
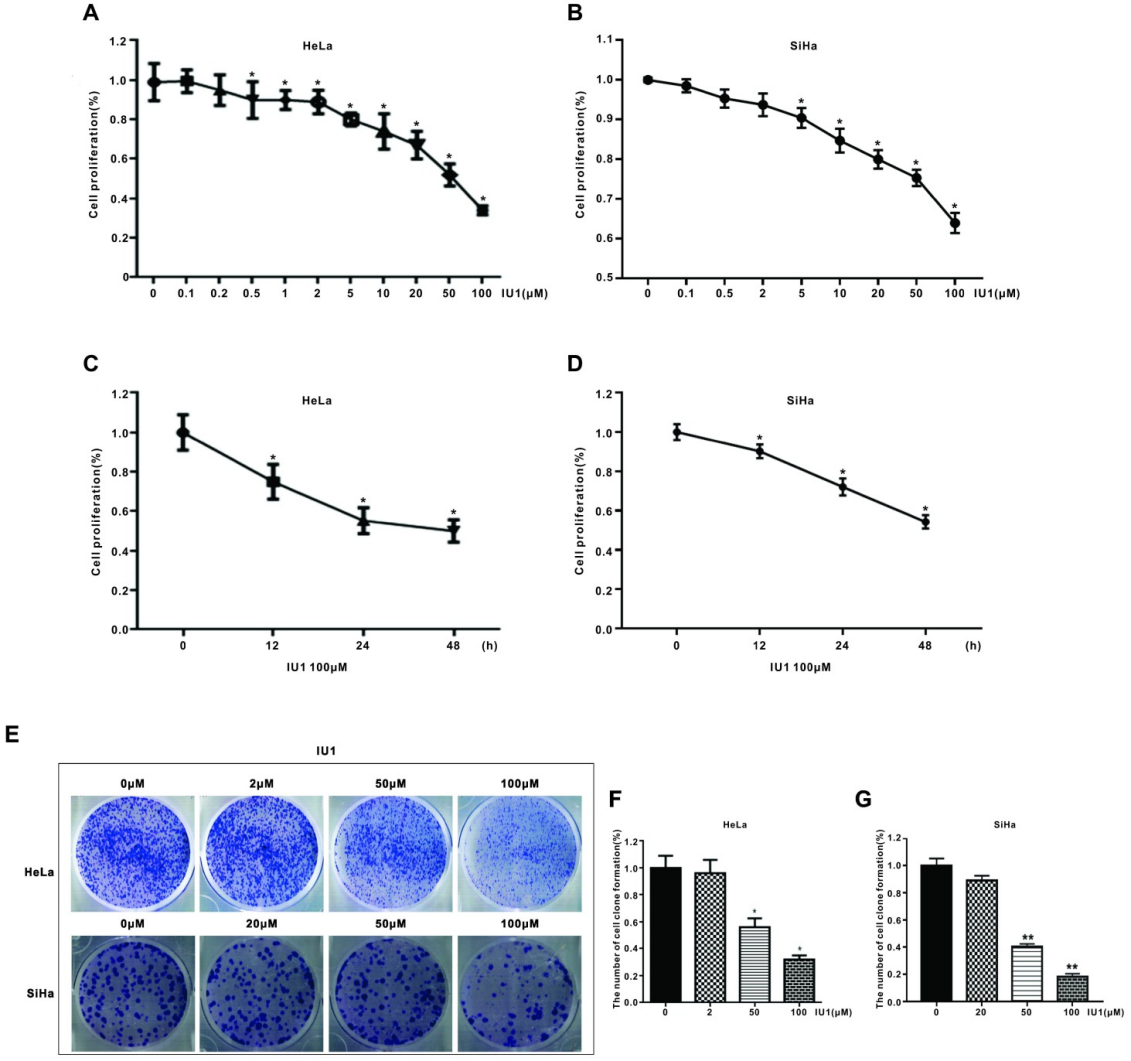
** IU1 suppresses the proliferation of cervical cancer cells. (A-B)** IU1 suppressed cervical cancer cell proliferation in dose-dependent manner. HeLa and SiHa cells were treated with a range of concentrations of IU1 for 24 h, as indicated. Cell viability was detected by CCK-8 assay. *p<0.05. **(C-D)** IU1 suppressed cervical cancer cell proliferation in time-dependent manner. HeLa and SiHa cells were treated with 100 µM IU1 for 12, 24, 48 h. Cell viability was detected by CCK-8 assay. *p<0.05. (**E**) Colony formation assay of cervical cells treated with IU1 or DMSO. HeLa and SiHa cells exposed to indicated concentrations of IU1 were suspended in 30% agarose for 1 week, representative images were shown. (**F-G**) The numbers of colonies were counted. Error bars correspond to 95% confidence intervals. *p<0.05, **p<0.01 compared with DMSO treatment.

Firstly, to determine the underlying effect of IU1 (a selective inhibitor of USP14) on the proliferation of cervical cancer cells, we used the CCK-8 assay. HeLa cells were treated with various concentrations of IU1 (0.1, 0.2, 0.5, 1, 2, 5, 10, 20, 50, 100 μM) for 24 h, or 100 μM of IU1 for 12, 24, 48 h. SiHa cells were treated with 0.1, 0.5, 2, 5, 10, 20, 50, 100 μM IU1 for 24h, or 100 μM of IU1 for 12, 24, 48 h. We found that IU1 significantly decreased cell proliferation in a time- and dose-dependent manner (Figure 1A-D). In order to test the long-term effect of IU1 treatment on cancer cells, we measured colony formation of HeLa cells using IU1 at 2, 50, 100 μM. SiHa cells were treated with 20, 50, 100 μM of IU1. As shown in Figure 1E-G, both treatment with IU1 50 μM and 100 μM dramatically decreased HeLa and SiHa cell colony formation after one week of culture.

### Effect of IU1 treatment on cell migration

**Figure 2:**
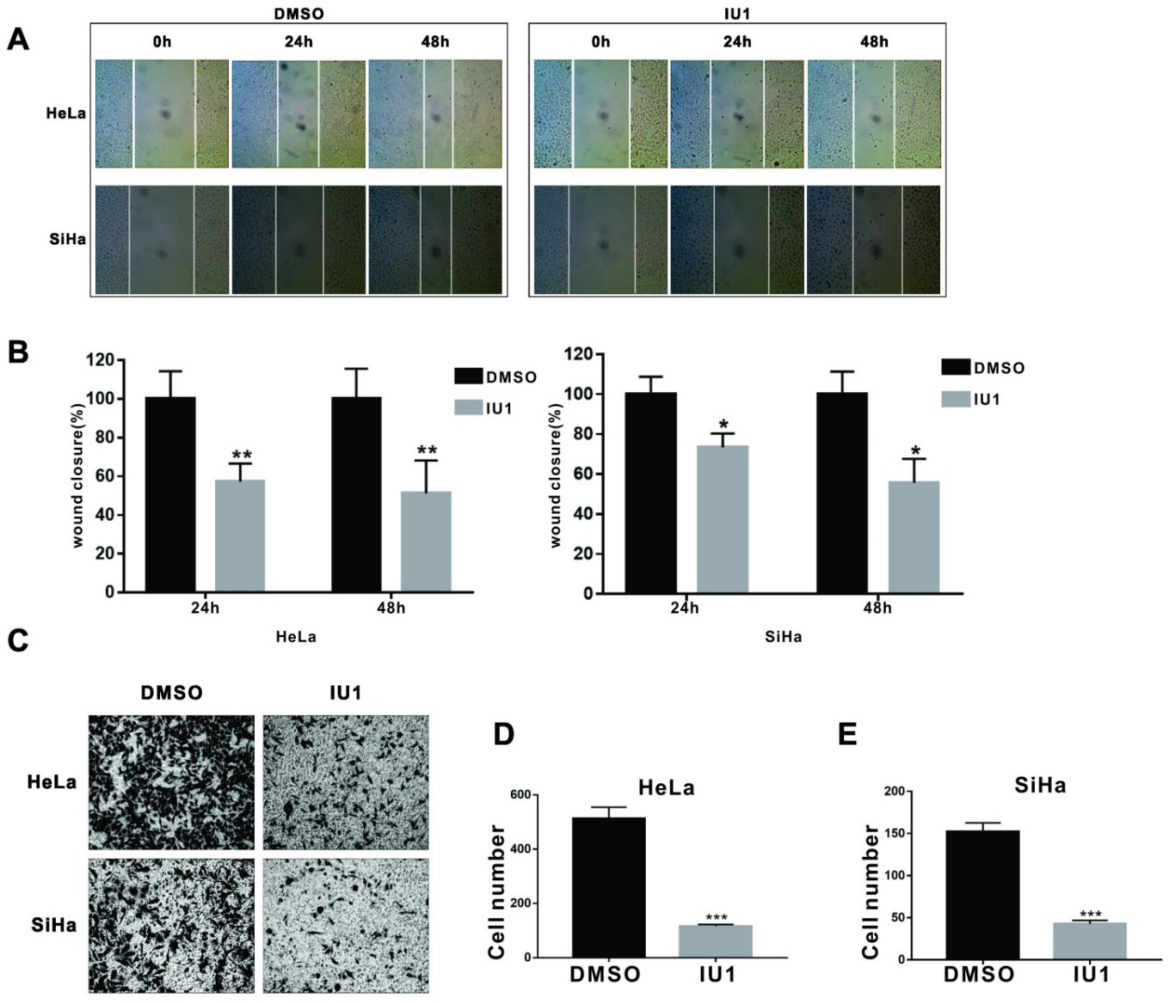
** IU1 impairs migration of cervical cancer cells. (A)** Wound healing assays were performed on HeLa and SiHa cells with 100 µM IU1. **(B)** Quantitative analysis of wound closure. **(C)** Boyden chamber method was used to examine the migration of HeLa and SiHa cells with 100 µM IU1 treatment for 36 h. **(D-E)** The number of cells migrating from the inside of the chamber to the outside were analysised in HeLa and SiHa cells. Data are means ± SEM. (n = 3). * *p*<0.05, **p<0.01, ***p<0.001 vs DMSO control.

Wound-healing assay was used to determine the impact of IU1 on cell migration. Monolayer HeLa and SiHa cells were grown to 100% confluence and then treated with 100 μM IU1 for 24 and 48 h. The results showed that the wound healing abilities of IU1-treated cells were markedly suppressed, as evidenced by the number of migrated cells in the closed area (Figure 2A and 2B). Furthermore, we examined the migration of HeLa and SiHa cells using the modified Boyden chamber method. The number of cells that migrated from the inside of the chamber to the outside was significantly decreased by IU1 treatment (100 μM) for 36 h in HeLa and SiHa cells (Figure 2C-E). These results suggest that IU1 treatment significantly suppress the migration of cervical cancer cells.

### IU1 treatment causes G_0_/G_1_ arrest and apoptosis in cervical cancer cells

**Figure 3:**
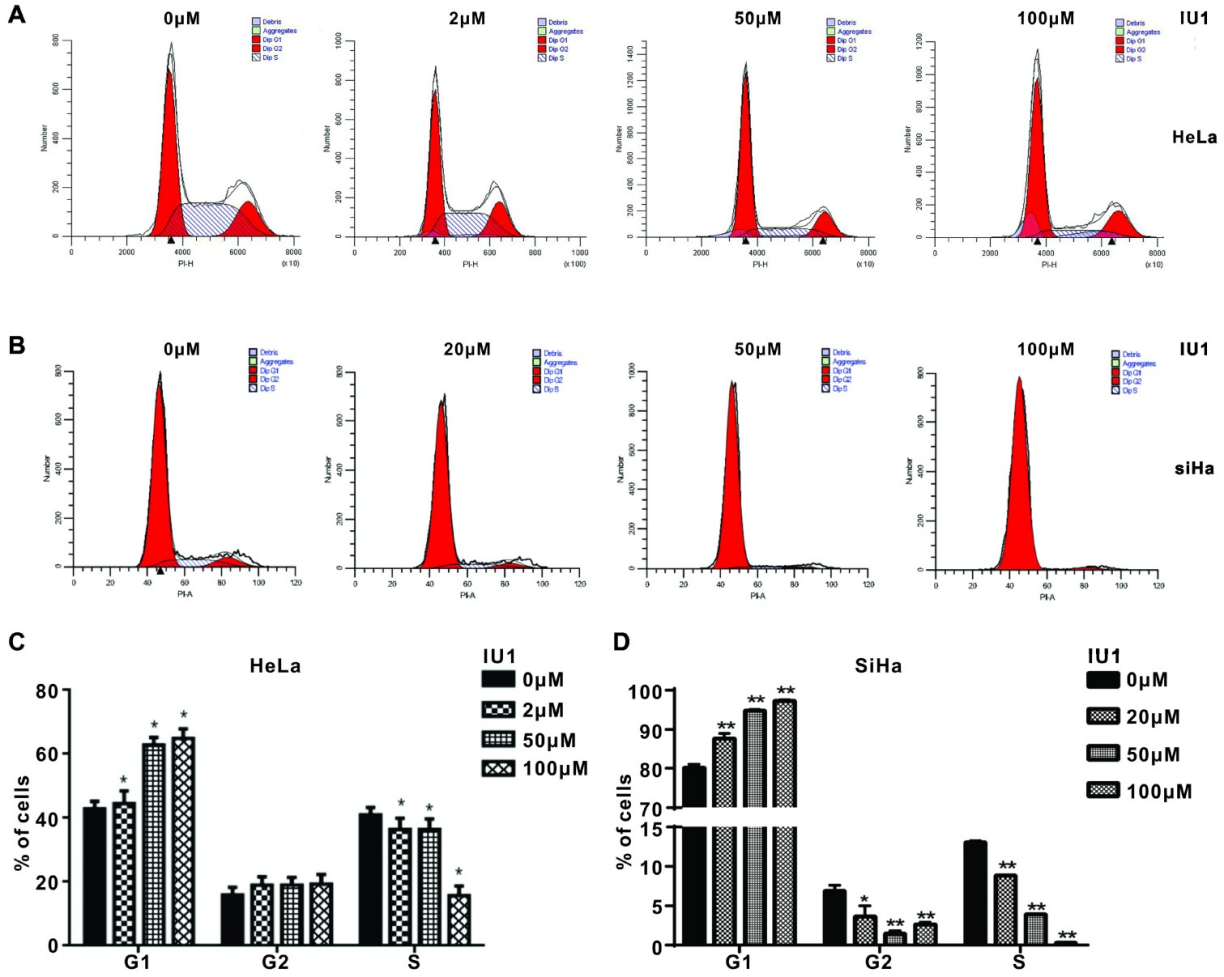
** IU1 blocks G0/G1 to S phase transition in cervical cancer cells. (A-B)** Shown are representative histograms of PI staining of HeLa and SiHa cells. Fluorescence activated cell sorting analysis were performed on HeLa and SiHa cells which exposed to the indicated concentrations of IU1 for 12 h. (**C-D**) The percentage of cells in each population as well as in each cell cycle phase at 12 h was calculated. *p<0.05, **p<0.01 compared with DMSO treatment.

We then studied the underlying mechanism by which IU1 regulates the growth of cervical cancer cells. We monitored the cell cycle progression of each group of HeLa cells exposed to various concentrations of IU1 (2, 50, 100 μM), or 20, 50, 100 μM of IU1 for SiHa cells. We found that IU1 treatment dramatically induced G_0_/G_1_ cell cycle arrest after 12 h, which was associated with the decreased population in S/G_2_/M phases (Figure 3A-D). Collectively, these results indicate that IU1 regulates the G_1_-S phase transition in cervical cancer cells.

**Figure 4:**
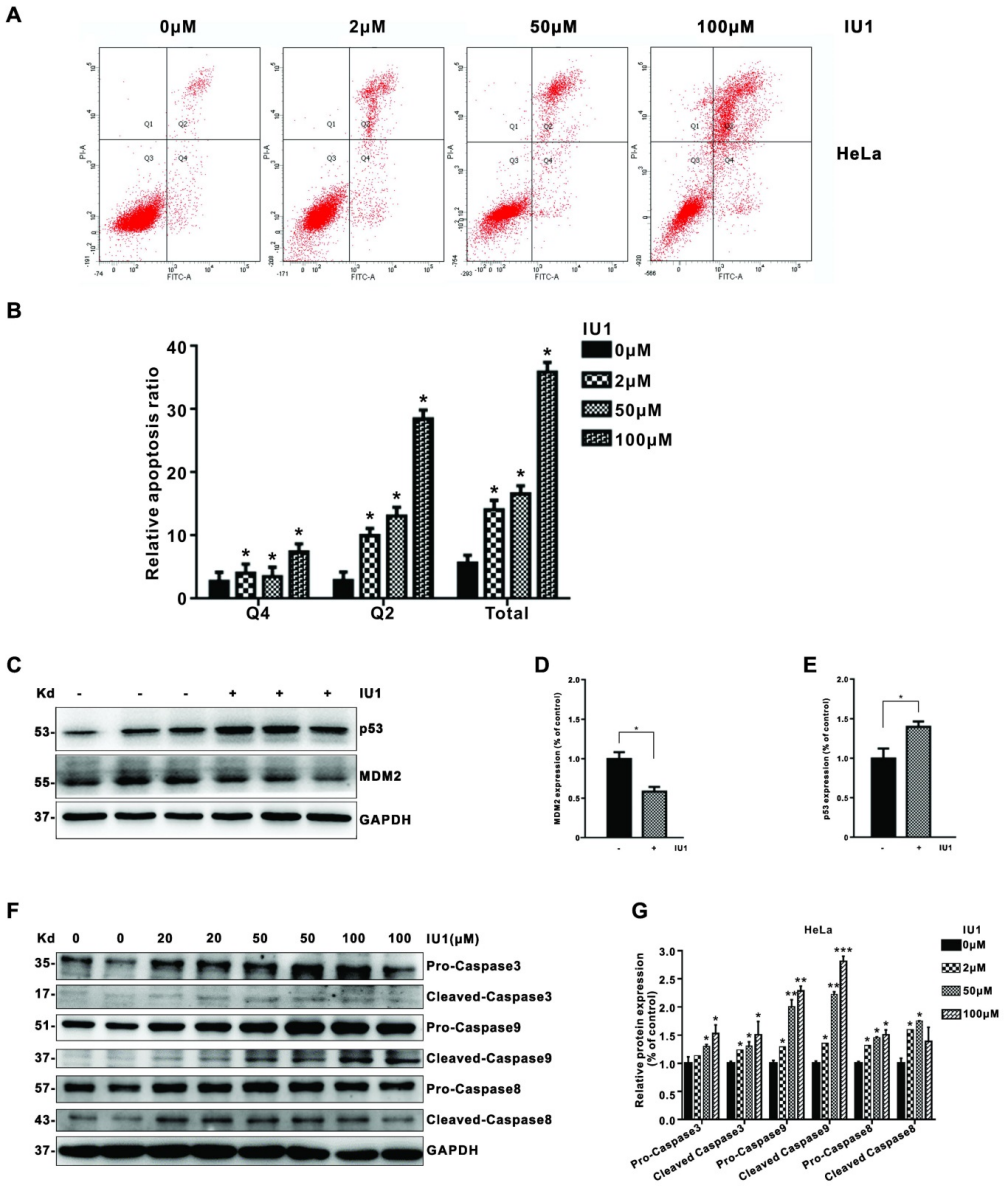
** IU1 induces cell apoptosis in cervical cancer cells. (A)** HeLa cells were treated with the different concentrations of IU1 for 12 h. The cultured cells were collected and stained with Annexin V-FITC/PI, followed by flow cytometry analysis. The representative images were shown. (**B**) The summary of relative cell apoptosis ratio was shown. **(C)** The expression of apoptosis-related proteins in IU1 treated HeLa cells were determined by western blot. HeLa cells were treated with 100 µM IU1 for 12 h. Total proteins were extracted and subjected to western blot analyses for MDM2 and p53. GAPDH was used as a loading control. **(D-E)** Quantification of MDM2 and p53 expression levels. IU1 inhibits the expression of MDM2, increases p53 levels in HeLa cells. **(F-G)** The protein expression levels of apoptosis-associated proteins (cleaved-caspase-3, cleaved-caspase-9, and cleaved-caspase-8) following IU1 treatment in HeLa cells were presented. Mean ± S.D. (n = 3). *p<0.05 compared with DMSO treatment.

We then determined whether apoptosis induction is also involved in the growth inhibition by IU1 treatment in cervical cancer cells. To do so, we further measured annexin V-fluorescein isothiocyanate (FITC)/propidium iodide (PI)-positive cells with flow cytometry. We found that IU1 treatment significantly induced apoptosis in HeLa cells (Figure 4A and 4B). Next, we determined the expression of apoptosis-related proteins by western blot to further explore the underlying mechanism of IU1 induced apoptosis. As shown in Figure 4C-E, the expression of MDM2 (murine double minute 2) clearly decreased after treatment with IU1 compared with the DMSO group (*p* < 0.05). By contrast, the expression levels of p53 were significantly upregulated in the IU1 group compared with the control (DMSO group) (*p* < 0.05). Taken together, these results indicate that IU1 promote HeLa cell apoptosis via regulating the expression of MDM2 and p53. We then validated the upregulation of apoptosis indicators following IU1 treatment, including cleaved caspase 3, cleaved caspase 9, and cleaved caspase 8. The expression levels of apoptosis-associated proteins were presented in Figure 4F and 4G. The expression levels of apoptosis-associated proteins (cleaved-caspase-3, cleaved-caspase-9, and cleaved-caspase-8) were increased significantly following IU1 treatment.

### IU1 treatment enhances the UPS function

**Figure 5:**
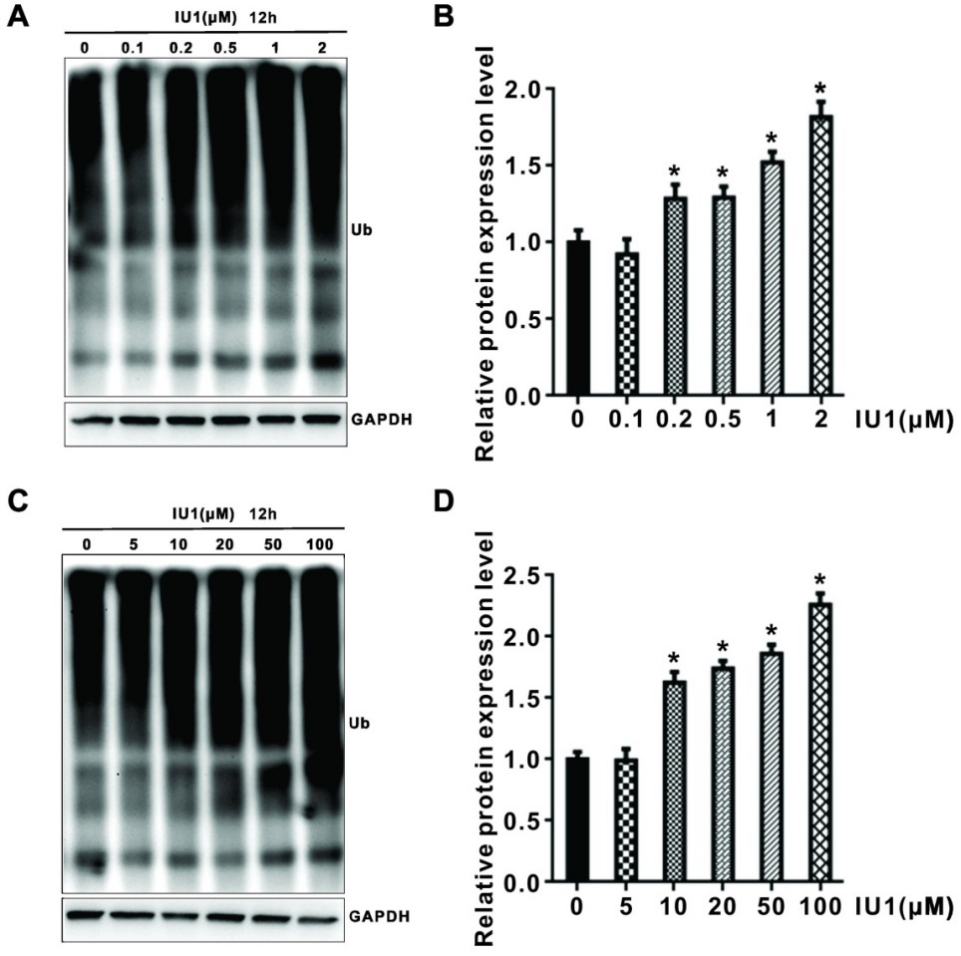
** Effect of IU1 on the ubiquitin proteasome system.** (**A** and **C**) Total protein ubiquitination. (**B** and **D**) Total protein ubiquitination was analyzed quantitativelyin a bar graph. Data are means ± SEM. (n = 3). *p<0.05 vs DMSO control.

To test whether degradation-specific UPS activity is increased in IU1-treated cells, HeLa cells were treated with 0.1, 0.2, 0.5, 1, 2, 5,10, 20, 50, and 100 μM IU1 for 3 h (Supplementary Figure S1) and 12 h (Figure 5A-D). Since that ubiquitination is one of the key regulatory steps for protein degradation, we further explored whether IU1 regulated total protein ubiquitination. As shown in Figure 5A-D, IU1 treatment for 12 h significantly enhanced total protein ubiquitination in HeLa cells in a dose-dependent manner. However, the total protein ubiquitination was not statistically significant in HeLa cells treated with IU1 for 3 h (Figure S1). These results indicate that IU1 treatment enhances UPS function at a late time-point.

### Effects of IU1 treatment on autophagy

**Figure 6:**
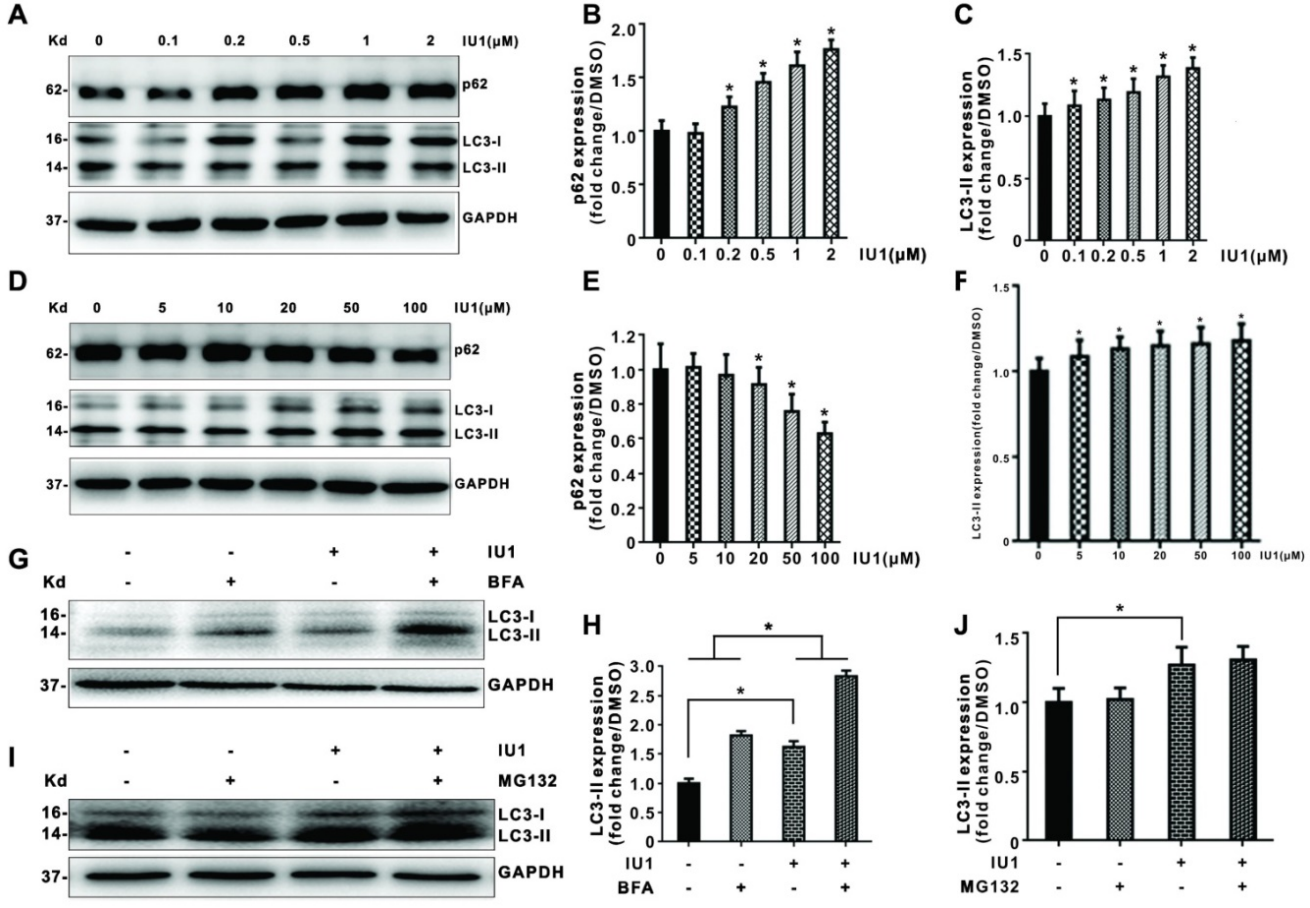
** Effect of IU1 on autophagy in HeLa cells. (A-F)** Protein expression levels of p62 and LC3 (autophagy marker). Panels are the representative images of western blot. The expression levels of p62 and LC3-II were analyzed quantitatively in a bar graph. **(G-J)** Autophagic flux. Panels are representative western blot bands of LC3. The expression levels of LC3-II were analyzed quantitatively in a bar graph. The quantitative analyses of autophagic flux were shown. Autophagic flux was estimated by calculating the difference in LC3-II turnover between the experimental groups. Data are means ± SEM. (n = 3). * *p*<0.05, ** *p*<0.01 vs DMSO-treated control.

**Figure 7:**
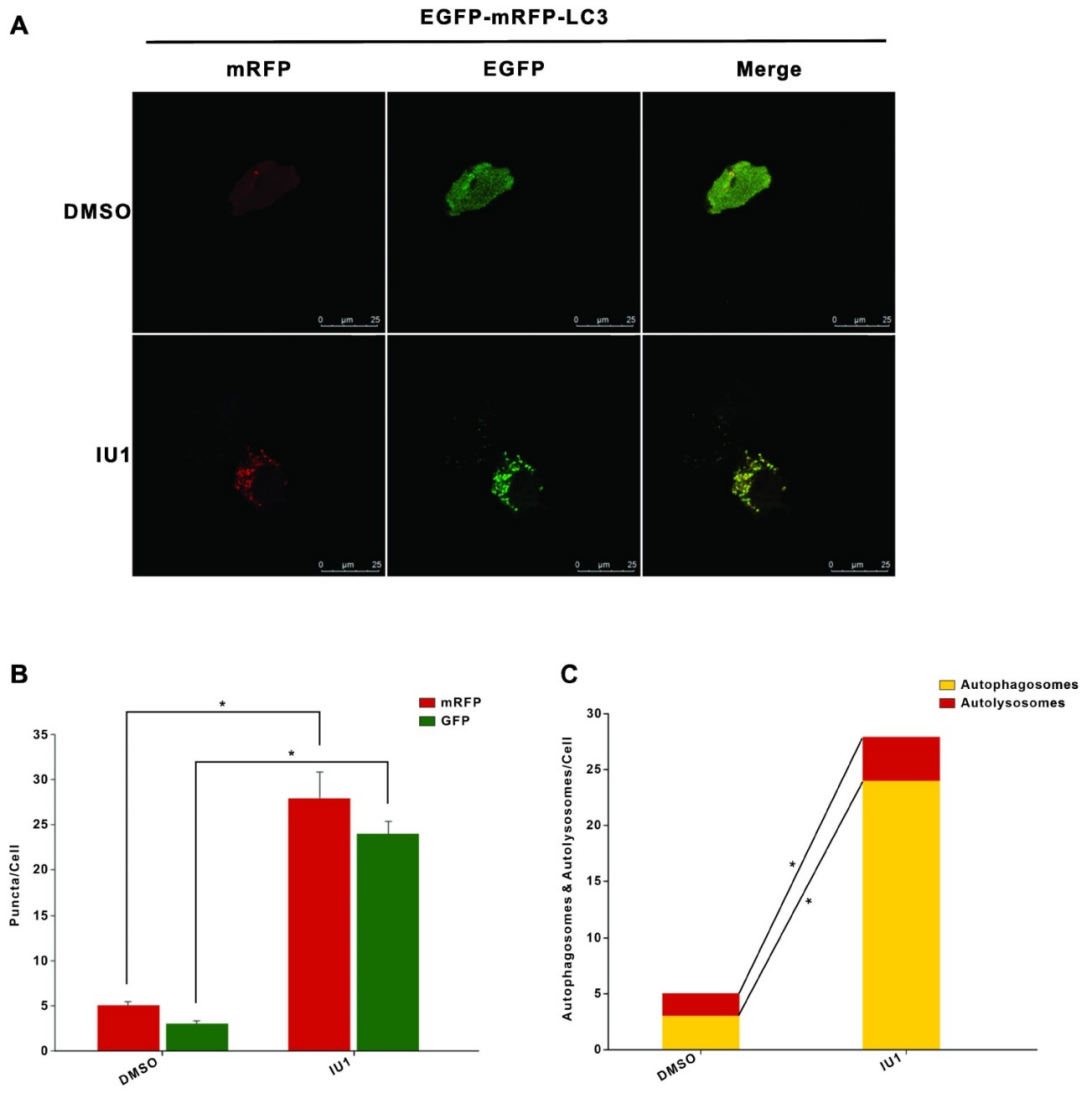
** The autophagy flux was induced when cells were treated with IU1. (A)** The EGFP-mRFP-LC3 assays *in vitro*. HeLa cells were transfected with pBabe-EGFP-mRFP-LC3 vector for 48 h and were subjected to IU1 100 µM for 12 h. Representative images of fluorescent LC3 puncta are shown. **(B)** Mean number of GFP and mRFP dots per cell. **(C)** Mean number of autophagosomes and autolysosomes per cell. Results represent the means from at least three independent experiments. *p < 0.05; **p< 0.01. Scale bar represents 25 µm.

To further verify the role of IU1 treatment in autophagy, we firstly investigate the effect of IU1 treatment on autophagy. HeLa cells were treated with 0.1, 0.2, 0.5, 1, and 2 μM IU1 for 12 h. The protein levels of LC3 and p62 (autophagy markers) were detected by western blot. As shown in Figure 6A-C, lower IU1 concentrations treatment dramatically increased the p62 protein levels and LC3-II expression in a dose-dependent manner. We further investigate the effect of higher IU1 concentrations treatment on autophagy. HeLa cells were treated with 5, 10, 20, 50, and 100 μM IU1 for 12 h. As shown in Figure 6D-F, higher IU1 concentrations treatment dramatically decreased p62 protein levels and increased LC3-II expression in a dose-dependent manner. To probe autophagic flux, HeLa cells were incubated with or without 100 μM IU1 for 12 h and then treated with 0.2 μM of autophagy inhibitor bafilomycin A (BFA) for 6 h. As expected, IU1 treatment significantly increased autophagy flux (Figure 6G and 6H). In contrast, treatment with MG132, an inhibitor of proteasomal degradation, did not affect LC3-II expression and autophagy flux (Figure 6I and 6J). Finally, when HeLa cells overexpressing pBabe-EGFP-mRFP-LC3 were treated with or without 100 μM IU1 for 12 h, we found that LC3 was distributed homogeneously in the cytoplasm and present yellow staining in untreated cells whereas the IU1 treatment significantly increased LC3 dots formation (Figure 7A-C). These results suggest that IU1 treatment activate autophagy.

### USP14 regulates MDM2 protein level in cervical cancer

**Figure 8:**
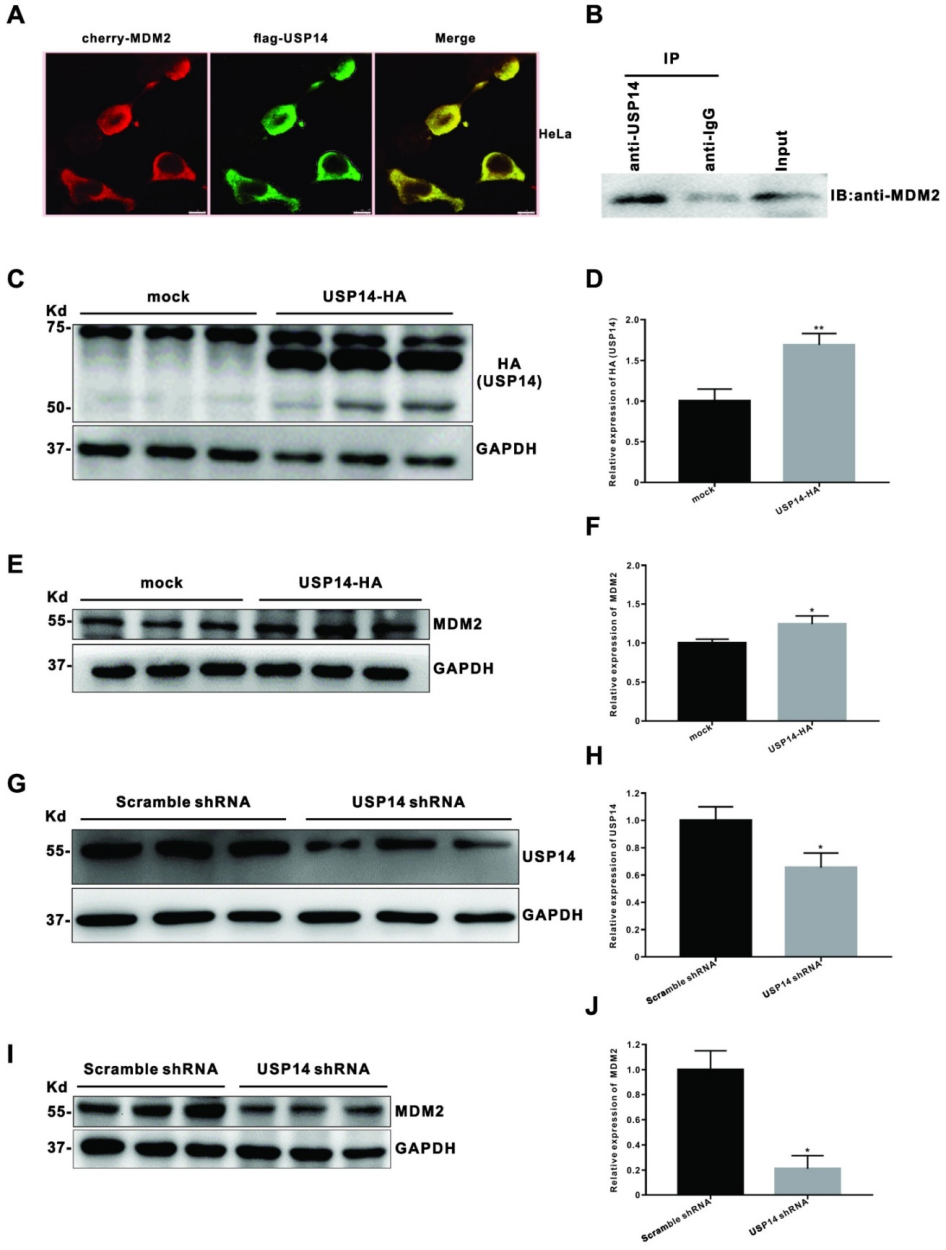
** USP14 interacts with MDM2 and upregulates MDM2 protein level. (A)** Immunofluorescent staining of GFP was executed after co-transfected with cherry-MDM2 and flag-USP14 plasmids for 24 h in HeLa cells. The co-localization between USP14 and MDM2 was revealed by immunofluorescent staining. **(B)** Co-immunoprecipitation (Co-IP) assay for the interaction between USP14 and MDM2 in HeLa cells. Total proteins were extracted from HeLa cells, immunoprecipitated with USP14 antibody beads and immunoblotted for MDM2. **(C-F)** After transfected with pLVX-puro vector or pLVX-USP14-HA for 48 h, the levels of HA (USP14) and MDM2 protein were detected by western blot in HeLa cells. **(G-J)** The HeLa or 293T cells were transfected with pLKO.1-shUSP14 or pLKO.1-EGFP-Puro vector (scramble shRNA control) for 48 h, and then the levels of USP14 and MDM2 protein were measured by western blot. *p < 0.05; **p< 0.01.

To explore the molecular mechanism by which IU1 regulates the growth of cervical cancer cells, we then query via https://bhapp.c2b2.columbia.edu/PrePPI/ and predict that USP14 and MDM2 can be combined. To test this hypothesis, we examined the MDM2 and p53 protein levels in HeLa cells exposed to 100 μM IU1 and found that IU1 decreased the protein levels of MDM2 (Figure 4C and 4D). Meanwhile, a moderate increase in p53 levels was observed (Figure 4C and 4E), which might be attributable to the decrease of MDM2. Moreover, co-immunoprecipitation assays showed that USP14 formed a complex with MDM2 (Figure 8B), which was consistent with the hypothesis that USP14 could bind to MDM2 and stabilize it. In addition, the co-localization between USP14 and MDM2 was revealed by immunofluorescent staining (Figure 8A), indicating that there was a possible interaction between the two molecules. To investigate whether USP14 overexpression could influence MDM2 protein degradation, HeLa cells were transiently transfected with a HA-tagged USP14 expression plasmid for 48 h and subjected to immunoblotting. As expected, we confirmed that MDM2 levels (Figure 8C-F) were increased in USP14-overexpressed cells compared to mock transfected HeLa cells. Conversely, to examine whether inhibition of USP14 could counteract MDM2 accumulation in cells, we then applied USP14 short hairpin RNA (shRNA) to knockdown USP14. We found that inhibition of USP14 expression caused the significant decrease in MDM2 expression levels in 293T cells. Collectively, these results showed that IU1 increased p53 expression via inhibiting MDM2 expression. Whereas MDM2 could interact with USP14, which removed the conjugated polyubiquitin chains from MDM2 and increased the stabilization of MDM2 protein.

## Discussion

Cervical cancer is the second most common type of cancer among women worldwide. Standard treatments have been found to be safe and effective. However, the clinical effects vary widely from one individual to the next. In China, about 130,000 new cases occur annually 1. It is considered a significant public health problem. Therefore, it is urgent to find new targets and drugs for the treatment of cervical cancer.

Recent studies have identified E3 ubiquitin ligase MDM2 as a novel therapeutic target in cervical cancer, unveiling a great treatment opportunity for cervical cancer patients 4, 19-23. MDM2, known as Murine double minute 2, is known to be a negative regulator of p53 tumor suppressor gene 22. MDM2 is made up of four functionally independent domains which include an N-terminal domain that recognizes the N-terminal Box-I domain of p53, and a RING finger domain critical for its E3 ubiquitin ligase activity. MDM2 has been extensively studied as an oncogene product. Aberrant MDM2 protein expression is documented in a wide variety of human cancers and is thought to be due to gene amplification as well as transcriptional and post-translational regulation 24-26. It is widely accepted that MDM2 mediated p53 ubiquitination and induced p53 degradation 22. Besides, MDM2 itself is the direct transcriptional target of p53. The interaction of MDM2 and p53 thereby forms an automatically feedback regulation loop that allows p53 and MDM2 to regulate each other's cellular levels and activities tightly. Small-molecule inhibitors of MDM2 blocking MDM2-p53 interaction or inhibiting ubiquitin ligase activity of MDM2 have been actively studied in advanced preclinical development or early-phase clinical trials for the treatment of cancer 2, 22, 23, 27, 28.

Our current study showed that IU1, a pharmacological deubiquitinating enzyme USP14 selective inhibitor, dramatically decreased MDM2 level, blocked G0/G1 to S phase transition, decreased cell growth and triggered cell apoptosis in cervical cancer cells, suggesting that targeting USP14/MDM2 axis could be a potential strategy for cervical cancer therapy.

We further explored the molecular mechanisms by which IU1 contributes to inhibiting cervical cancer development and progression. The intracellular protein levels are regulated by processes including transcription, translation, and protein degradation. Our data showed that IU1 treatment did not affect mRNA expression of MDM2 (data not shown), which suggests that IU1 regulates MDM2 protein levels by a post-translational mechanism.

Evidences have shown the involvement of UPS in MDM2 degradation 7, 8, 29-32. Because of that ubiquitination is one of the key regulatory steps for protein degradation, we further explored whether total protein ubiquitination was regulated by IU1. In the present study, our data have shown that IU1 treatment significantly increased the total protein ubiquitination at a late time-point, which indicated that IU1 treatment promoted UPS function.

Another major clearance route for intracellular protein is autophagy. Autophagy exerts tumor suppressor effect in normal cells. Autophagy disorders may lead to malignant transformation of cells and carcinogenesis. In tumors, autophagy is believed to promote tumor growth and progression by helping cells adapt to the harsh tumor microenvironment. Therefore, some autophagy inhibitors, such as hydroxychloroquine (HCQ), are used in anti-cancer treatment strategies 16. Recent studies have found that IU1 could induce autophagy in cancer cells 15. Our results showed that IU1 treatment significantly increased protein expression of autophagy marker LC3-II, as well as EGFP-LC3 puncta formation which suggests that LC3 is recruited to the autophagosomal membrane during autophagosome formation. With the addition of increased autophagy flux, our data strongly suggested that IU1 activated autophagy. Thus, the present studies support the notion that IU1 induces both autophagy and UPS-dependent MDM2 degradation by which IU1 exerts its antitumor effects.

DUBs have emerged as a class of novel therapeutic targets or biomarkers for antitumor strategies. Besides DUB inhibitor IU1 discussed above, USP7/HAUSP inhibitors are the most fascinating DUB inhibitor due to its role in regulating p53 function. P5091, as an inhibitor of USP7, actively inhibits USP7, resulting in increased steady-state protein levels of p53 and p21. Moreover, WP1130, which is found to be a partially selective DUB inhibitor, directly inhibits DUB activity of USP9x, USP5 and USP14 33-37. Notably, several DUBs have been reported to regulate MDM2 expression or transcriptional activity 8, 29-32, 38. As an example, it was reported in the literature that USP15 acted as a deubiquitinase of MDM2, which bound to MDM2 and directly cleaved the ubiquitin chains from MDM2. USP15 stabilized MDM2 and regulated p53 function in cancer cells. In colorectal cell lines, loss of USP15 caused ubiquitination and degradation of MDM2, which indicated that the amount of MDM2 is maintained by the dynamic balance between ubiquitination and deubiquitination 10. In addition, USP7 is also involved in the regulation of MDM2 in cancer cells. USP7 was initially identified as a DUB that stabilized p53, but subsequent studies had shown that USP7 also regulated the amount of MDM2 in cancer cells 39, 40. USP14 and USP7 have the same domain and similar functions. Therefore, our current study investigated the roles of USP14 for the regulation of MDM2. It was predicted that USP14 and MDM2 could be combined via the https://bhapp.c2b2.columbia.edu/PrePPI/ query. We found that USP14 and MDM2 were co-localized and directly interacted, which was confirmed with co-IP. In this study, we suggest that USP14, as an MDM2 DUB, is required to remove the ubiquitin chain from MDM2 and stabilize MDM2 protein.

The interaction between the ubiquitin-proteasome system (UPS) and autophagy in one pathway ultimately affects another. Drugs that act on only one of the systems may result in treatment failure due to the compensatory effects of another system 41. The treatment failure demonstrates the disadvantages of using UPS and autophagy as isolated systems and highlights the importance of conducting these two systems as collaborative and complementary systems. USP14 is involved in both proteasome and autophagy-mediated protein degradation, and plays an essential role in this interaction system 42, 43. Our study reveals that IU1 simultaneously targets UPS and autophagy systems, inhibits tumor cell proliferation, and promotes tumor cell apoptosis in cervical cancer cells.

In conclusion, this work has provided a novel insight into the interaction between proteasome-associated DUB USP14 and MDM2 in cervical cancer cells. Furthermore, the current study proposes a potential antitumor mechanism of IU1 and broadens clinical application prospects for the treatment of reproductive system tumors.

## Supplementary Material

Supplementary figures and tables.

## Abbreviations

USP14: deubiquitinase ubiquitin-specific protease 14
UPS: ubiquitin-proteasome system
MDM2: Murine double minute 2
DUBs: Deubiquitinating Enzymes
GFAP: glial fibrillary acidic protein
MEFs: mouse embryonic fibroblasts
FITC: fluorescein isothiocyanate
BFA: bafilomycin A

## References

[1] National Health Commission Of The People's Republic Of C (2019). Chinese guidelines for diagnosis and treatment of cervical cancer 2018 (English version). Chin J Cancer Res.

[2] Gupta A, Shah K, Oza MJ, Behl T (2019). Reactivation of p53 gene by MDM2 inhibitors: A novel therapy for cancer treatment. Biomed Pharmacother.

[3] Li J, Kurokawa M (2015). Regulation of MDM2 Stability After DNA Damage. J Cell Physiol.

[4] Yuan Y, Liao YM, Hsueh CT, Mirshahidi HR (2011). Novel targeted therapeutics: inhibitors of MDM2, ALK and PARP. J Hematol Oncol.

[5] Devine T, Dai MS (2013). Targeting the ubiquitin-mediated proteasome degradation of p53 for cancer therapy. Curr Pharm Des.

[6] An T, Gong Y, Li X, Kong L, Ma P, Gong L (2017). USP7 inhibitor P5091 inhibits Wnt signaling and colorectal tumor growth. Biochem Pharmacol.

[7] Lee G, Oh TI, Um KB, Yoon H, Son J, Kim BM (2016). Small-molecule inhibitors of USP7 induce apoptosis through oxidative and endoplasmic reticulum stress in cancer cells. Biochem Biophys Res Commun.

[8] Zhang W, Sartori MA, Makhnevych T, Federowicz KE, Dong X, Liu L (2017). Generation and Validation of Intracellular Ubiquitin Variant Inhibitors for USP7 and USP10. J Mol Biol.

[9] Zhou J, Wang J, Chen C, Yuan H, Wen X, Sun H (2018). USP7: Target Validation and Drug Discovery for Cancer Therapy. Med Chem.

[10] Zou Q, Jin J, Hu H, Li HS, Romano S, Xiao Y (2014). USP15 stabilizes MDM2 to mediate cancer-cell survival and inhibit antitumor T cell responses. Nat Immunol.

[11] Lee BH, Lee MJ, Park S, Oh DC, Elsasser S, Chen PC (2010). Enhancement of proteasome activity by a small-molecule inhibitor of USP14. Nature.

[12] Homma T, Ishibashi D, Nakagaki T, Fuse T, Mori T, Satoh K (2015). Ubiquitin-specific protease 14 modulates degradation of cellular prion protein. Sci Rep.

[13] Kiprowska MJ, Stepanova A, Todaro DR, Galkin A, Haas A, Wilson SM (2017). Neurotoxic mechanisms by which the USP14 inhibitor IU1 depletes ubiquitinated proteins and Tau in rat cerebral cortical neurons: Relevance to Alzheimer's disease. Biochim Biophys Acta Mol Basis Dis.

[14] Boselli M, Lee BH, Robert J, Prado MA, Min SW, Cheng C (2017). An inhibitor of the proteasomal deubiquitinating enzyme USP14 induces tau elimination in cultured neurons. J Biol Chem.

[15] Xia X, Huang C, Liao Y, Liu Y, He J, Guo Z (2019). Inhibition of USP14 enhances the sensitivity of breast cancer to enzalutamide. J Exp Clin Cancer Res.

[16] Mooneyham A, Bazzaro M (2017). Targeting Deubiquitinating Enzymes and Autophagy in Cancer. Methods Mol Biol.

[17] Pal A, Young MA, Donato NJ (2014). Emerging potential of therapeutic targeting of ubiquitin-specific proteases in the treatment of cancer. Cancer Res.

[18] Sontag EM, Vonk WIM, Frydman J (2014). Sorting out the trash: the spatial nature of eukaryotic protein quality control. Curr Opin Cell Biol.

[19] Bohlman S, Manfredi JJ (2016). Mdm2-RNA Interactions as a Target for Cancer Therapy: It's Not All About p53. Cancer Cell.

[20] Makii C, Oda K, Ikeda Y, Sone K, Hasegawa K, Uehara Y (2016). MDM2 is a potential therapeutic target and prognostic factor for ovarian clear cell carcinomas with wild type TP53. Oncotarget.

[21] Gordon EM, Ravicz JR, Liu S, Chawla SP, Hall FL (2018). Cell cycle checkpoint control: The cyclin G1/Mdm2/p53 axis emerges as a strategic target for broad-spectrum cancer gene therapy - A review of molecular mechanisms for oncologists. Mol Clin Oncol.

[22] Chene P (2003). Inhibiting the p53-MDM2 interaction: an important target for cancer therapy. Nat Rev Cancer.

[23] Shaikh MF, Morano WF, Lee J, Gleeson E, Babcock BD, Michl J (2016). Emerging Role of MDM2 as Target for Anti-Cancer Therapy: A Review. Ann Clin Lab Sci.

[24] Du W, Yi Y, Zhang H, Bergholz J, Wu J, Ying H (2013). Rapamycin inhibits IGF-1-mediated up-regulation of MDM2 and sensitizes cancer cells to chemotherapy. PLoS One.

[25] Agrawal A, Yang J, Murphy RF, Agrawal DK (2006). Regulation of the p14ARF-Mdm2-p53 pathway: an overview in breast cancer. Exp Mol Pathol.

[26] Wang G, Firoz EF, Rose A, Blochin E, Christos P, Pollens D (2009). MDM2 expression and regulation in prostate cancer racial disparity. Int J Clin Exp Pathol.

[27] Zhao Y, Aguilar A, Bernard D, Wang S (2015). Small-molecule inhibitors of the MDM2-p53 protein-protein interaction (MDM2 Inhibitors) in clinical trials for cancer treatment. J Med Chem.

[28] Gu L, Zhang H, Liu T, Zhou S, Du Y, Xiong J (2016). Discovery of Dual Inhibitors of MDM2 and XIAP for Cancer Treatment. Cancer Cell.

[29] Bhattacharya S, Chakraborty D, Basu M, Ghosh MK (2018). Emerging insights into HAUSP (USP7) in physiology, cancer and other diseases. Signal Transduct Target Ther.

[30] Colland F, Formstecher E, Jacq X, Reverdy C, Planquette C, Conrath S (2009). Small-molecule inhibitor of USP7/HAUSP ubiquitin protease stabilizes and activates p53 in cells. Mol Cancer Ther.

[31] Shi Y, Solomon LR, Pereda-Lopez A, Giranda VL, Luo Y, Johnson EF (2011). Ubiquitin-specific cysteine protease 2a (USP2a) regulates the stability of Aurora-A. J Biol Chem.

[32] Turnbull AP, Ioannidis S, Krajewski WW, Pinto-Fernandez A, Heride C, Martin ACL (2017). Molecular basis of USP7 inhibition by selective small-molecule inhibitors. Nature.

[33] Wang Z, Kang W, You Y, Pang J, Ren H, Suo Z (2019). USP7: Novel Drug Target in Cancer Therapy. Front Pharmacol.

[34] Farshi P, Deshmukh RR, Nwankwo JO, Arkwright RT, Cvek B, Liu J (2015). Deubiquitinases (DUBs) and DUB inhibitors: a patent review. Expert Opin Ther Pat.

[35] Garcia DA, Baek C, Estrada MV, Tysl T, Bennett EJ, Yang J (2018). USP11 Enhances TGFbeta-Induced Epithelial-Mesenchymal Plasticity and Human Breast Cancer Metastasis. Mol Cancer Res.

[36] Gavory G, O'Dowd CR, Helm MD, Flasz J, Arkoudis E, Dossang A (2018). Discovery and characterization of highly potent and selective allosteric USP7 inhibitors. Nat Chem Biol.

[37] Islam MT, Zhou X, Chen F, Khan MA, Fu J, Chen H (2019). Targeting the signalling pathways regulated by deubiquitinases for prostate cancer therapeutics. Cell Biochem Funct.

[38] Sun XX, Challagundla KB, Dai MS (2012). Positive regulation of p53 stability and activity by the deubiquitinating enzyme Otubain 1. EMBO J.

[39] Tavana O, Gu W (2017). Modulation of the p53/MDM2 interplay by HAUSP inhibitors. J Mol Cell Biol.

[40] Sun Y, Cao L, Sheng X, Chen J, Zhou Y, Yang C (2017). WDR79 promotes the proliferation of non-small cell lung cancer cells via USP7-mediated regulation of the Mdm2-p53 pathway. Cell Death Dis.

[41] Chude CI, Amaravadi RK (2017). Targeting Autophagy in Cancer: Update on Clinical Trials and Novel Inhibitors. Int J Mol Sci.

[42] Min Y, Lee S, Kim MJ, Chun E, Lee KY (2017). Ubiquitin-Specific Protease 14 Negatively Regulates Toll-Like Receptor 4-Mediated Signaling and Autophagy Induction by Inhibiting Ubiquitination of TAK1-Binding Protein 2 and Beclin 1. Front Immunol.

[43] Xu D, Shan B, Sun H, Xiao J, Zhu K, Xie X (2016). USP14 regulates autophagy by suppressing K63 ubiquitination of Beclin 1. Genes Dev.

