# Supplementary material for: IU1 suppresses proliferation of cervical cancer cells through MDM2 degradation
Source: Int J Biol Sci. 2020 Sep 16;16(15):2951–63. doi: 10.7150/ijbs.47999 (PMC7545697; doi:10.7150/ijbs.47999)
Supplement: Supplementary file 1 — Supplementary figures and tables. [file ijbsv16p2951s1.pdf]

**Figure S1. Effect of IU1 on the ubiquitin proteasome system for 3 h. (A)** Total protein ubiquitination. **(B-C)** Total protein ubiquitination were analyzed quantitatively in a bar graph. Data are means ± SEM. (n = 3). *$p$<0.05 vs DMSO control.

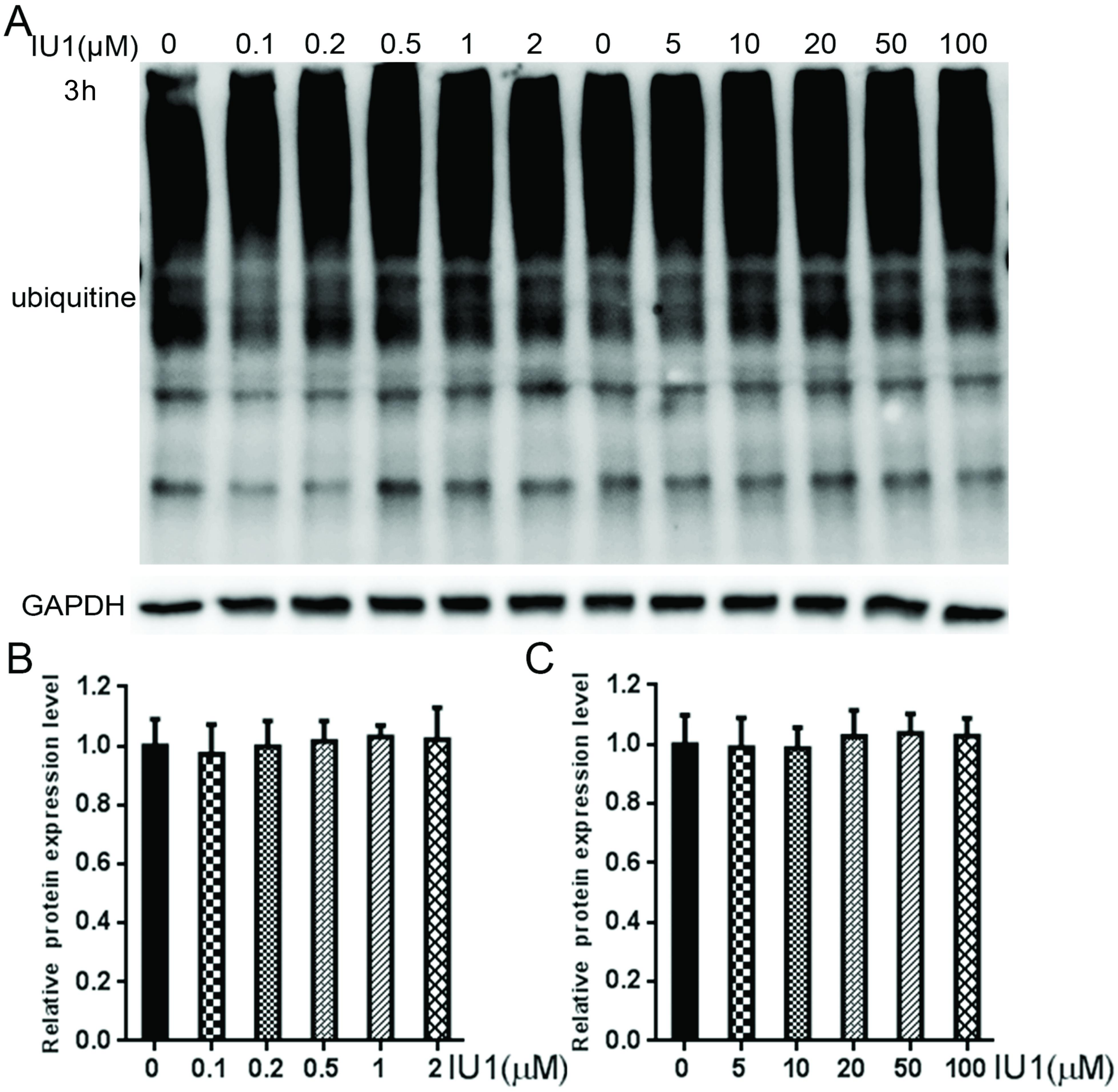

A
IU1(μM) 0 0.1 0.2 0.5 1 2 0 5 10 20 50 100
3h
ubiquitine
GAPDH
B
Relative protein expression level
1.2
1.0
0.8
0.6
0.4
0.2
0.0
0 0.1 0.2 0.5 1 2 IU1(μM)
C
Relative protein expression level
1.2
1.0
0.8
0.6
0.4
0.2
0.0
0 5 10 20 50 100 IU1(μM)
